# Correction: Empirical estimates of the mutation rate for an alphabaculovirus

**DOI:** 10.1371/journal.pgen.1010383

**Published:** 2022-09-01

**Authors:** The PLOS Genetics Staff

## Notice of republication

This article was republished on July 12, 2022, to remove an erroneous ORCID iD. The publisher apologizes for the errors. Please download this article again to view the correct version.
