# Supplementary figures and images for: Empirical estimates of the mutation rate for an alphabaculovirus
Source: PLoS Genet. 2022 Jun 6;18(6):e1009806. doi: 10.1371/journal.pgen.1009806 (PMC9203023; doi:10.1371/journal.pgen.1009806)

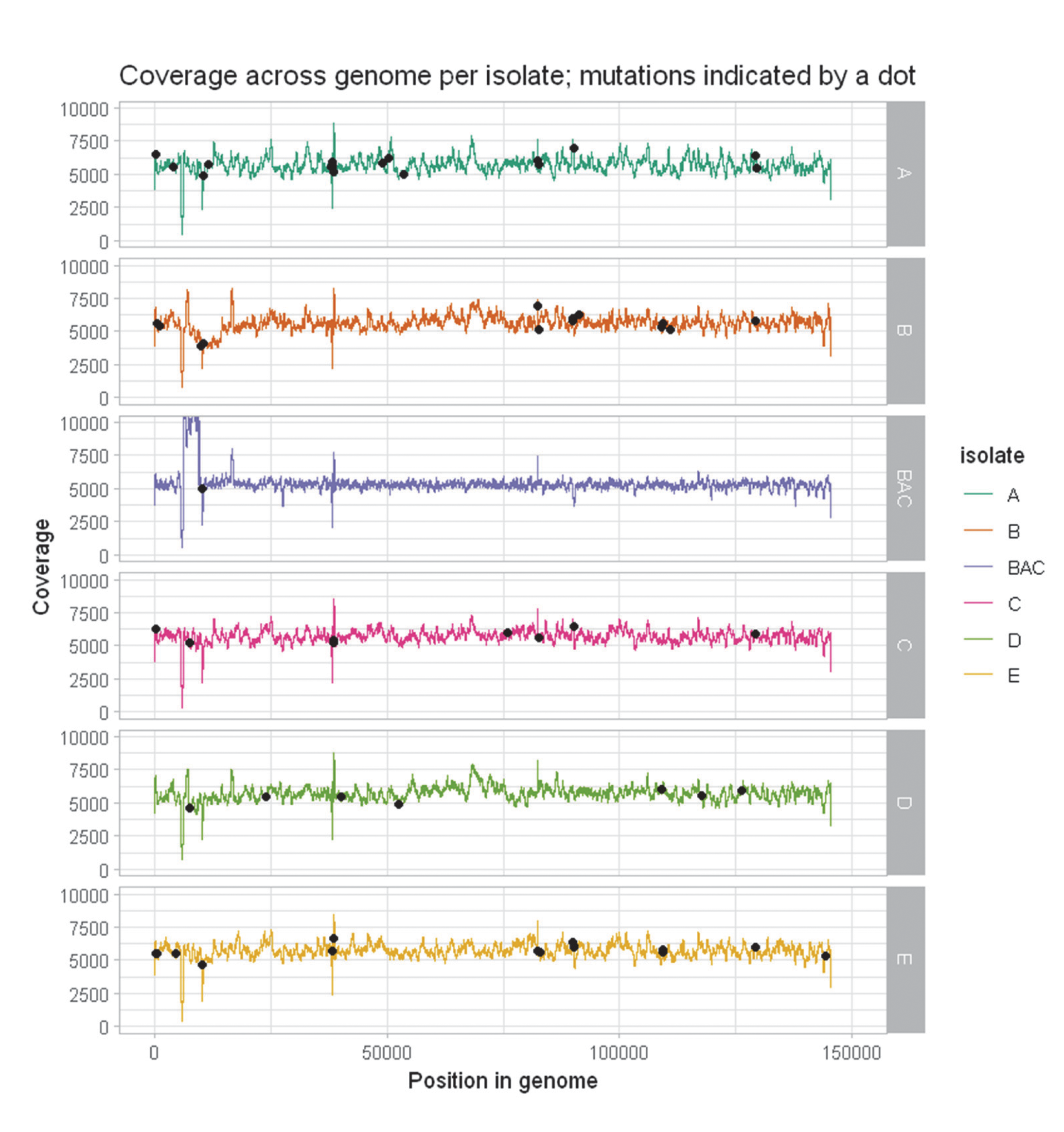

Supplement: S1 Fig — We show coverage along the genome for each evolved line (A, B, C, D and E) as well as ancestral strain BAC. Position of mutations observed at mutation frequency threshold value (τ) = 0.5 and present only in a single evolved population (ψ = 1) are shown as black dots. Coverage patterns are similar between the different isolates. The peak observed at around 10000 bp for the BAC isolate is due to the presence of empty bacmid vectors in sequencing data and is omitted from mutation calling. (TIF) [file pgen.1009806.s001.tif]

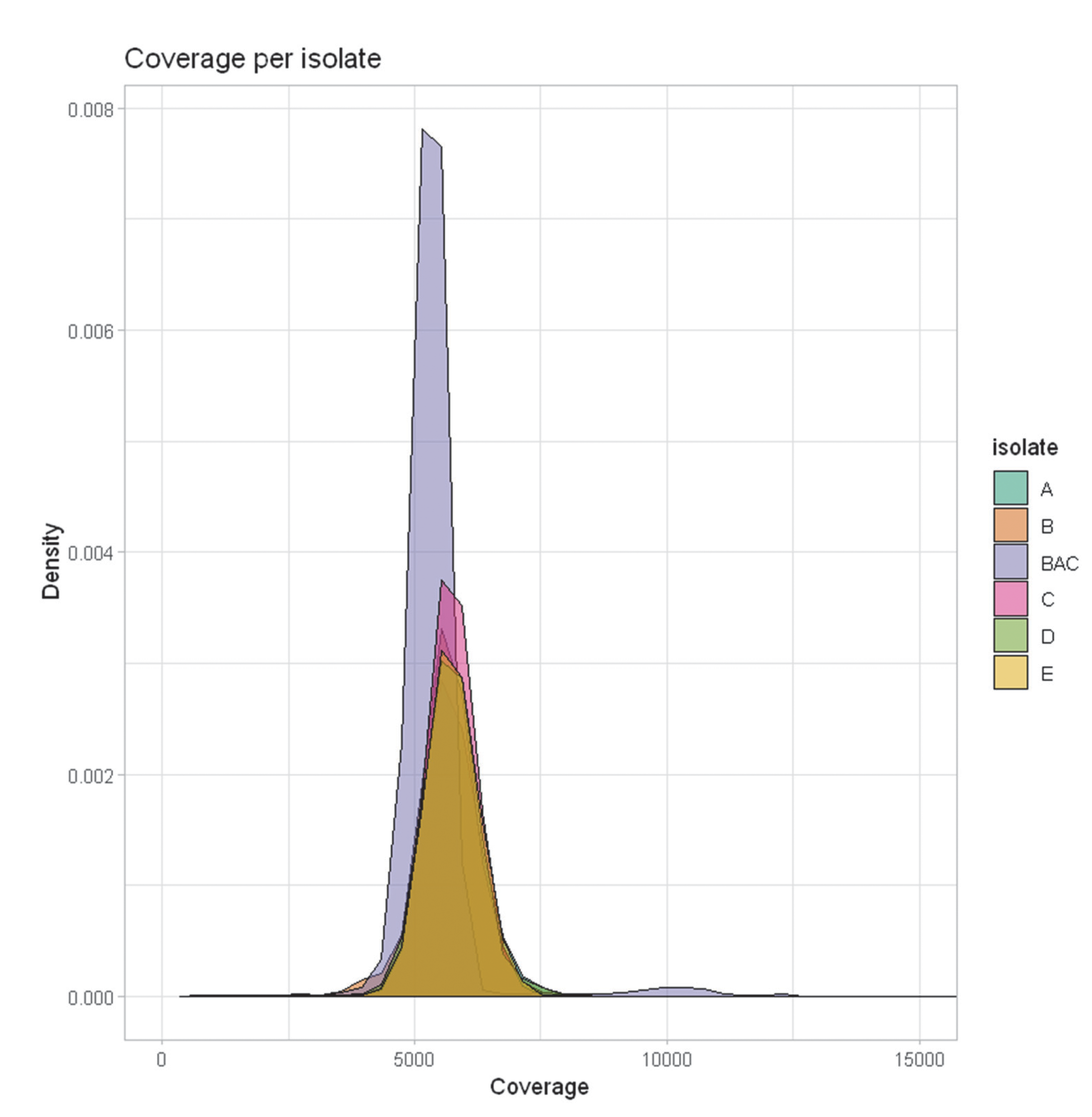

Supplement: S2 Fig — Isolates have a mean coverage of around 5500. The BAC isolate is showing an additional peak at a coverage of around 10000, which is explained by the presence of empty bacmid vectors in sequencing data. (TIF) [file pgen.1009806.s002.tif]

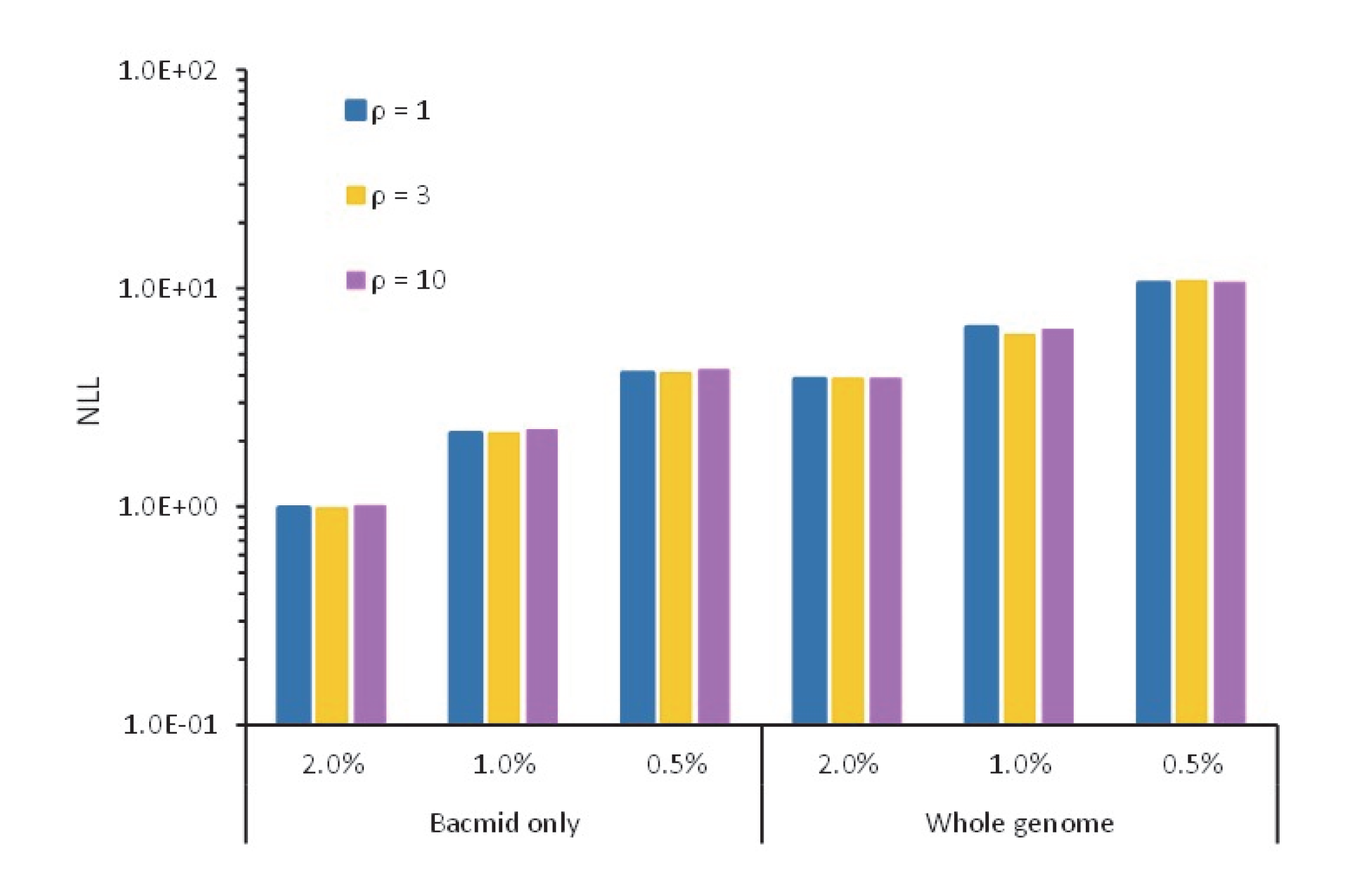

Supplement: S3 Fig — For simplicity, we show the results when only unique mutations are considered (ψ = 1). Mutation frequency threshold values clearly effect model fit, as they have an effect on the number of mutations that will be detected. By contrast, assumptions on the value for the parameter that determines the mode of virus replication (ρ) had little effect on model fit. This result is not surprising however, given that our model does not consider the frequency of mutations, but simply the number of bases with a mutation frequency greater than τ. (TIF) [file pgen.1009806.s003.tif]

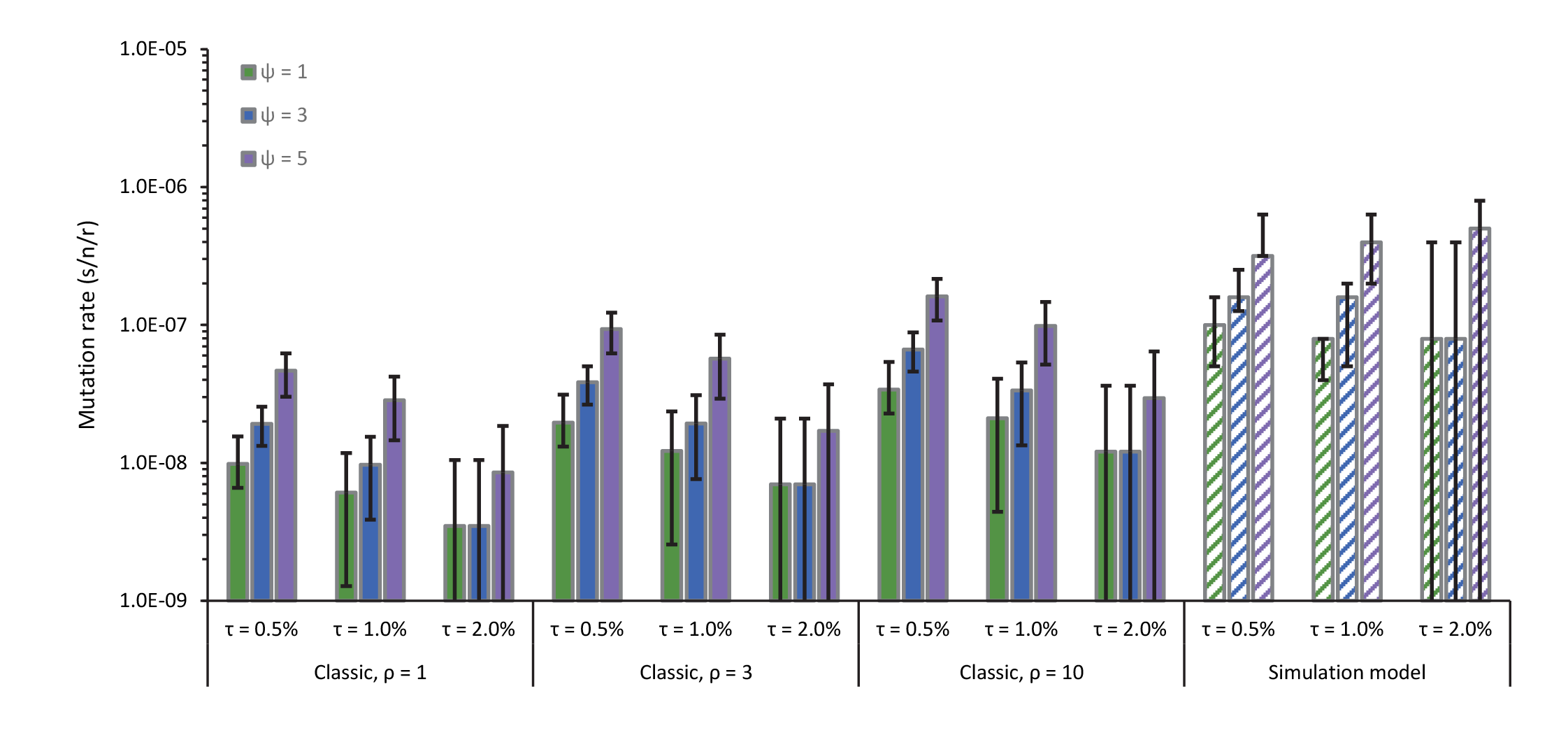

Supplement: S4 Fig — Mutation rates were estimated for different values of the viral mode of replication (⍴), different values for the threshold of mutation detection (τ), and different values for of the maximum number of lineages in which a mutation could occur before being excluded from the analysis (ψ). Error bars represent the 95% fiducial limits, as determined by bootstrapping. When the lower fiducial limit extends beyond the lower limit of the axis, this indicates a lower fiducial limit of zero. Overall, these estimates were lower than those obtained with the approach employing a simulation model. As baculoviruses most likely employ rolling circle amplification, replication is likely to have a high value of ⍴. Therefore, the best estimates with this approach assume the highest value of ⍴. Moreover, they will assume the lowest mutation detection threshold (τ), provided all mutations are assumed to be bona fide, as the cumulative frequency of mutations above this value is used required to estimate mutations and no correction is made for this threshold. Finally, as in our other analyses, we think the most conservative estimate of mutation rate will exlude all repeated mutations (ψ = 1). These conditions (⍴ = 10, τ = 0.5%, ψ = 1) render an estimate of μ = 3 x 10−8 s/n/r, which is lower but roughly similar to for our simulation-based approach (μ ~ 10−7). (TIF) [file pgen.1009806.s004.tif]

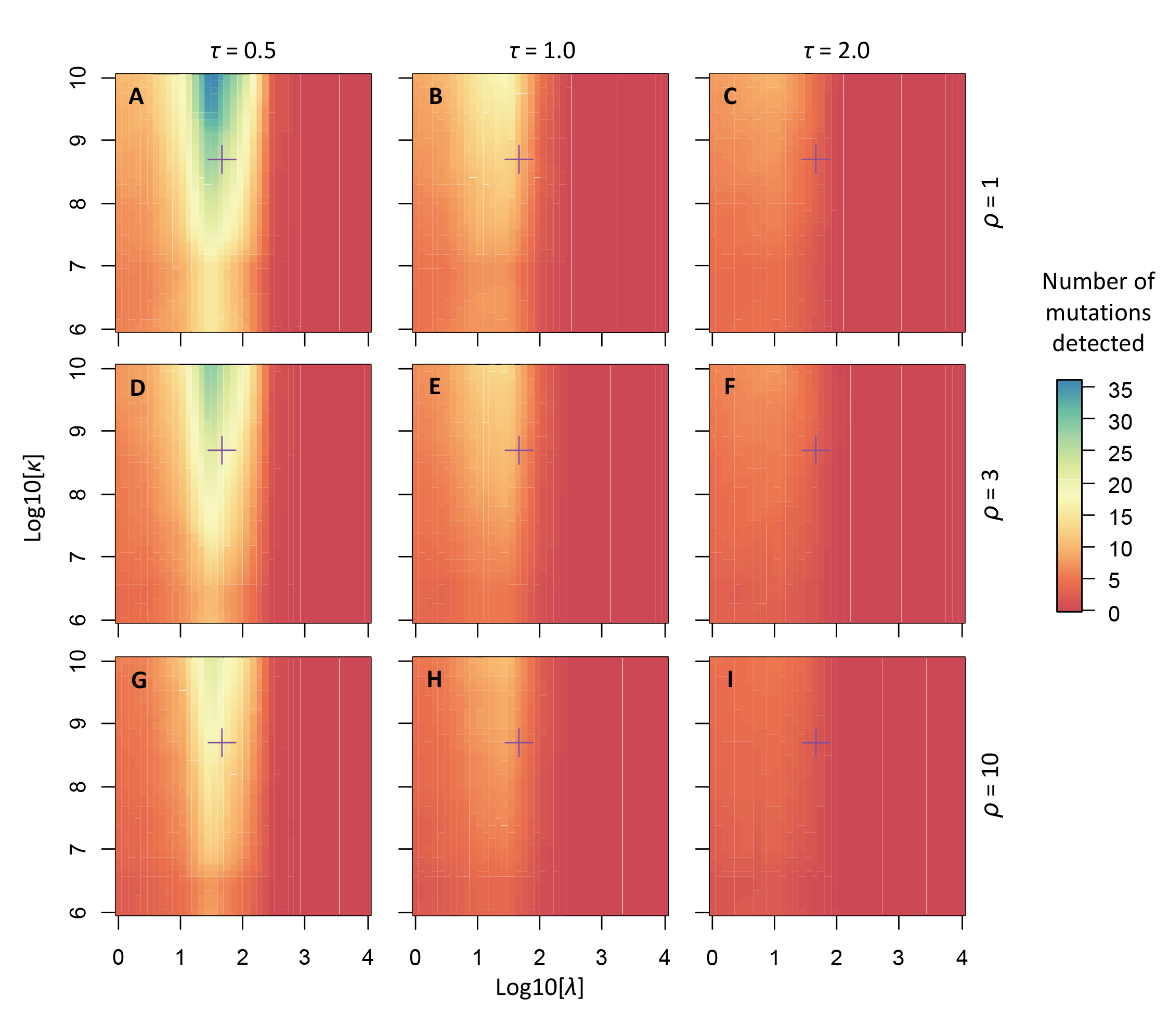

Supplement: S5 Fig — For all simulations, we assumed a mutation rate similar to our estimated value for baculoviruses (μ = 10−7), and kept other model parameters the same as for model fitting (Table 4) unless otherwise indicated. We varied the size of the founding viral population in one insect (λ, x-axis is the log10[λ]) and the final size of the viral population in one insect (κ, y-axis is the log10[κ]), while also varying the threshold value for mutation detection (τ) and mode of virus replication (ρ) over the different panels. The purple cross indicates the point in the parameter space that corresponds to the model parameters assumed in model fitting (λ = 46, κ = 5.05 × 10^8^). There are more detectable mutations when τ is low, when ρ is low, and as the final population size κ increases. Increases in the size of the founding viral population λ initially lead to increasing numbers of detectable mutations, but the number of detectable mutations eventually decreases. For an explanation of this non-monotonic behaviour, see S6 Fig. Finally, note that we can also predict mutation accumulation using the established approach (see Materials and Methods Section) for comparison purposes, which does not take τ into account. The range of model predictions for the number of accumulating mutations (lowest to highest predicted value, based on the extreme values of λ and κ) is then: for ρ = 1, 0.48–2.42 mutations; for ρ = 3, 0.24–1.21 mutations; for ρ = 10, 0.13–0.70 mutations. The simulation model which takes into consideration better the effects of demography on mutation accumulation, therefore predicts considerably lower and higher mutation accumulation under some conditions. (TIF) [file pgen.1009806.s005.tif]

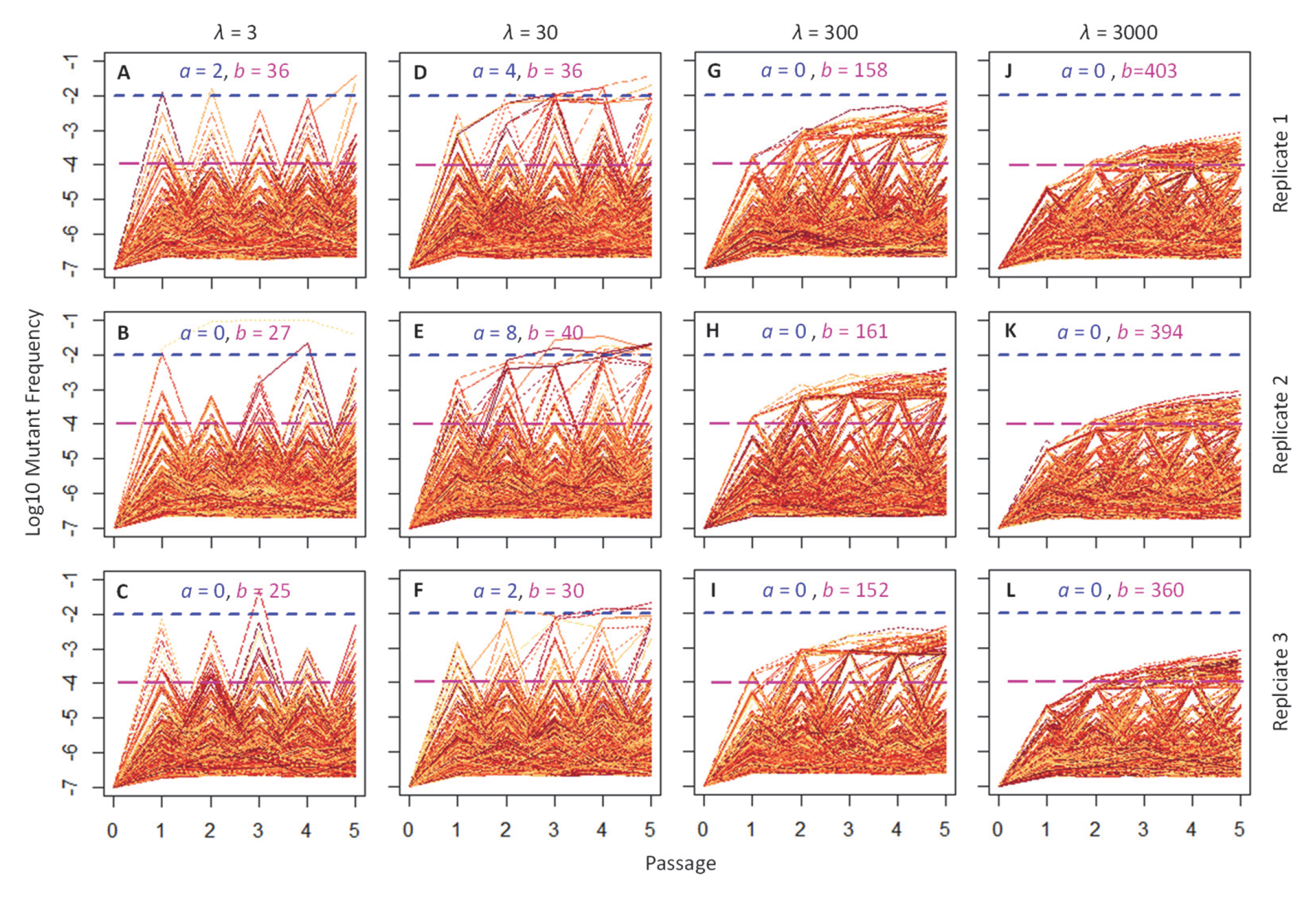

Supplement: S6 Fig — The simulation model was run for 5 passages in single insect larvae, with a genome size of g = 50,000 base pairs, mutation rate μ = 10−7 and final population size κ = 3 × 10^8^. We then varied λ, as indicated at the top of each column of panels, with all panels in a column simply representing replicate simulations. We plotted of the log10-transformed frequency of mutations at each position (y-axis) at the end of each round of passaging (x-axis), randomly selecting a hue and line type for each position to make them easier to distinguish. Finally, for each panel we noted the number of mutations which were above a frequency of 0.01 (a, with the threshold indicated by a blue line) and mutations above a frequency of 0.0001 (b, with the threshold indicated by a purple line). We assume the a mutations will be detected by sequencing, as a ~ τ, the threshold value for mutation detection used. The b mutations are sometimes maintained in the population over passages, but they need not be detected as they can be below τ. The number of b mutations increases as λ is increased, whereas the number of a mutations only increases initially. Wide bottlenecks will lead to the maintenance of more mutations in the population, but they also limit the stochastic increases in mutation frequency and prevent mutations from reaching the detection threshold. Recall that all mutations are assumed to be strictly neutral, and that all changes in mutation in mutation frequency are due to de novo mutations or genetic drift. (TIF) [file pgen.1009806.s006.tif]
