# Supplementary material for: Empirical estimates of the mutation rate for an alphabaculovirus
Source: PLoS Genet. 2022 Jun 6;18(6):e1009806. doi: 10.1371/journal.pgen.1009806 (PMC9203023; doi:10.1371/journal.pgen.1009806)
Supplement: S1 Data — (PDF) [file pgen.1009806.s011.pdf]

Workflow_10_2020

Workflow Input (FastBacDual_polh_1805_annotated)	
Workflow Input	FastBacDual_polh_1805_annotated
Import Command	

Map Reads to Reference	
References	Defined by: Workflow Input (FastBacDual_polh_1805_annotated)
Masking mode	No masking
Masking track	
Match score	1
Mismatch cost	2
Cost of insertions and deletions	Linear gap cost
Insertion cost	3
Deletion cost	3
Insertion open cost	6
Insertion extend cost	1
Deletion open cost	6
Deletion extend cost	1
Length fraction	0.5
Similarity fraction	0.8
Global alignment	false
Auto-detect paired distances	true
Non-specific match handling	Map randomly

Low Frequency Variant Detection	
Required significance (%)	1.0
Ignore positions with coverage above	100000
Restrict calling to target regions	
Ignore broken pairs	true
Ignore non-specific matches	Reads
Minimum read length	20
Minimum coverage	10
Minimum count	2
Minimum frequency (%)	0.5

Low Frequency Variant Detection	
Base quality filter	true
Neighborhood radius	5
Minimum central quality	20
Minimum neighborhood quality	15
Read direction filter	false
Direction frequency (%)	5.0
Relative read direction filter	true
Significance (%)	1.0
Read position filter	false
Significance (%)	1.0
Remove pyro-error variants	false
In homopolymer regions with minimum length	3
With frequency below	0.8

Trim Reads	
Quality trim	true
Quality limit	0.05
Ambiguous trim	true
Ambiguous limit	2
Trim adapter list	
Automatic read-through adapter trimming	true
Trim homopolymers from 5'	false
Trim homopolymers from 3'	false
polyA	false
polyC	false
polyG	true
polyT	false
Remove 5' terminal nucleotides	false
Number of 5' terminal nucleotides	1
Remove 3' terminal nucleotides	false
Number of 3' terminal nucleotides	1
Fixed length trimming	false
Maximum length	150
Trim from side	3'-end

Trim Reads	
Discard short reads	true
Minimum number of nucleotides in reads	15
Discard long reads	true
Maximum number of nucleotides in reads	1000

QC for Read Mapping	
Long contigs threshold	10000
Short contigs threshold	200
