# Supplementary material for: Empirical estimates of the mutation rate for an alphabaculovirus
Source: PLoS Genet. 2022 Jun 6;18(6):e1009806. doi: 10.1371/journal.pgen.1009806 (PMC9203023; doi:10.1371/journal.pgen.1009806)
Supplement: S1 File — PDF file with supplementary tables, including Table A (Analysis of the distribution of mutations along the genome), Table B (Relative frequencies of mutations, ψ = 1), Table C (Relative frequencies of mutations, ψ = 3), and Table D (Relative frequencies of mutations, ψ = 5). (PDF) [file pgen.1009806.s016.pdf]

S1 File: Supplementary Tables A-D

Table A File S1: Analysis of the distribution of mutations along the genome. These analyses were performed for the lowest mutation frequency threshold (τ = 0.5%) to ensure sufficient mutations for a meaningful analyses. The variable ψ indicates the maximum number of evolved lineages in which a mutation can occur and still be accepted. Mutations indicates the number of unique mutation positions analyzed, *D* is the test statistic for the Kolmogorov-Smirnoff test, and P-value indicates significance for a two-sided comparison of the observed mutations to uniform distribution across the region under consideration. P-values below a threshold value of 0.05 are indicated with *, and values below 0.001 are indicated with ***.

Region	ψ	Mutations	*D*	P-value
Bacmid	1	8	0.4410	0.062
	3	13	0.4412	0.008*
	5	20	0.4681	< 0.001***
Viral genome	1	67	0.2100	0.005*
	3	116	0.2359	< 0.001***
	5	146	0.2518	< 0.001***

Table B File S1: Relative frequencies of mutations observed per type, for the whole genome and bacmid only datasets, across mutation frequency threshold values (τ, indicated as a percentage), for mutations that occur only in a single evolved population (ψ = 1).

	Relative frequency of mutation				
	Whole genome			Bacmid only	
Mutation	τ = 0.5 (n=68)	τ = 1 (n=20)	τ = 2 (n=8)	τ = 0.5 (n=8)	Mutation type
CA	12	5	1	1	Transversion
CG	1	0	0	0	Transversion
TA	3	0	0	0	Transversion
TG	1	0	0	1	Transversion
AC	3	0	0	1	Transversion
AT	4	0	0	1	Transversion
GC	2	0	0	0	Transversion
GT	15	6	1	1	Transversion
GA	9	4	2	2	Transition
CT	6	3	2	0	Transition
TC	5	1	1	0	Transition
AG	7	1	1	1	Transition

Table C File S1: Relative frequencies of mutations observed per type, for the whole genome and bacmid only datasets, across mutation frequency threshold values (*τ*, indicated as a percentage), for mutations that occur in up to three evolved populations (*ψ* = 3).

	Relative frequency of mutation				
	Whole genome			Bacmid only	
Mutation	τ = 0.5 (n=180)	τ = 1 (n=44)	τ = 2 (n=9)	τ = 0.5 (n=17)	Mutation type
CA	20	9	1	4	Transversion
CG	1	0	0	0	Transversion
TA	21	7	1	0	Transversion
TG	4	0	0	1	Transversion
AC	12	2	0	1	Transversion
AT	16	3	0	1	Transversion
GC	4	0	0	0	Transversion
GT	24	10	1	3	Transversion
GA	19	4	2	2	Transition
CT	18	4	2	0	Transition
TC	18	2	1	1	Transition
AG	23	3	1	4	Transition

Table D File S1: Relative frequencies of mutations observed per type, for the whole genome and bacmid only datasets, across mutation frequency threshold values (*τ*, indicated as a percentage), for mutations that occur in up to five evolved populations (ψ = 5).

	Relative frequency of mutation				
	Whole genome			Bacmid only	
Mutation	τ = 0.5 (n=281)	τ = 1 (n=83)	τ = 2 (n=15)	τ = 0.5 (n=37)	Mutation type
CA	24	12	1	4	Transversion
CG	6	2	0	0	Transversion
TA	41	20	2	4	Transversion
TG	4	0	0	1	Transversion
AC	14	2	0	1	Transversion
AT	26	7	0	5	Transversion
GC	8	1	1	0	Transversion
GT	29	10	1	3	Transversion
GA	27	8	3	10	Transition
CT	29	6	2	0	Transition
TC	33	10	4	1	Transition
AG	40	5	1	8	Transition
