# Supplementary material for: Empirical estimates of the mutation rate for an alphabaculovirus
Source: PLoS Genet. 2022 Jun 6;18(6):e1009806. doi: 10.1371/journal.pgen.1009806 (PMC9203023; doi:10.1371/journal.pgen.1009806)
Supplement: S1 Text — (PDF) [file pgen.1009806.s019.pdf]

S1 Text

Supplementary Text 1: Relevance of viral demography for mutation rate estimates

We used predictions from a simulation model to estimate mutation rates, for which we estimate the mutation rate (μ) and the viral replication mode (ρ) parameters. As model fitting required considerable computational resources, we fixed all model parameters for which we could deduce values from our experimental observations or the literature (see Materials and Methods section). However, to gauge how model parameters affected predictions, we considered the effects of two key demographic parameters: the virus population bottleneck size in each insect (λ) and the final size of the virus population within each insect (κ) on mutation accumulation. We considered these effects concurrently with variation in τ (threshold frequency for mutation detection) and ρ (model of virus replication). The model was run with the mutation rate fixed to a relevant value ($\mu = 10^{-7}$), and we considered the mean number of mutations that accumulated after five passages equivalent to the experimental setup (i.e., in pool of 5 individual insect per passage, but for simplicity without a final amplification step). We found that all these parameters had an effect on mutation accumulation, within some regions of the parameter space (Figure S5). This result emphasizes the relevance of using models that incorporate virus demographics for estimating mutation rates, as well the importance of having robust estimates of these parameters when this is possible. Our estimates of λ and κ are both good approximations based on empirical data (see Materials and Methods), whereas we are not aware of estimates ρ for baculoviruses and the choice of τ is a methodological consideration.

Surprisingly, the model predicted that for wide bottlenecks ($\lambda > 100$), no mutations would not be detected after passaging, irrespective of the values of the other parameters tested (Figure S5). To understand this outcome better, we considered the frequency of mutations at all positions over passages for different bottleneck sizes. As the bottleneck becomes wider, more mutations are maintained in the population over time (Figure S6). However, wide

bottlenecks also prevent neutral mutations from rapidly reaching the threshold frequency required for mutation detection (i.e., τ values of ≥ 0.5%). To explain this effect intuitively, consider that a narrow bottleneck of $\lambda = 1$ will immediately fix any mutations present in the variant sampled. By contrast, for a wide bottleneck of $\lambda = 1000$, the large number of individuals sampled will result in a founding population that is more representative of the inoculum and genetic drift can only lead to small increases in the frequency of a rare variant. This result further stresses the importance of considering demographic processes when estimating viral mutation rates based on mutation accumulation; virus populations often pass through narrow, dose-dependent bottlenecks (See reference 42 in main manuscript, Zwart & Elena, 2015).
