# Supplementary material for: Empirical estimates of the mutation rate for an alphabaculovirus
Source: PLoS Genet. 2022 Jun 6;18(6):e1009806. doi: 10.1371/journal.pgen.1009806 (PMC9203023; doi:10.1371/journal.pgen.1009806)
Supplement: Attachment — Submitted filename: Response to reviewers.pdf [file pgen.1009806.s020.pdf]

We thank the reviewers for their constructive comments on this study. In the table below, we give the reviewer comments and our responses for each reviewer. Minor comments, as flagged by the reviewers, have been shaded in light grey for easy reference. In the manuscript file, all major changes or new text have been highlighted in yellow.

Reviewer's comment	Author's response
Reviewer 1	
The authors argue that the low copy number bacterial insert can be considered as neutral, and provide evidence of this neutral character in Figure 3 by comparing the rate of synonymous versus non synonymous mutations in this regions compared to the genuine baculovirus genes. For the former, there is conclusion of neutrality, while for the later there is a global conclusion of purifying selection. As not all virus genes are selected at the same level, it could be of interest to differentiate them into "highly selected and loosely selected". However, is the number of mutations enough for that approach to be carried out?	In response to other reviewer comments, we revised our approach for filtering mutations to make them less stringent. Even so, using the least stringent criteria used for filtering mutations (detection threshold τ = 0.5%, mutations repeated in up to 5 lineages accepted), we have only 23 unique nonsynonymous mutations in the viral genome, as many of mutations found are synonymous or in intergenic regions. This number is indeed too low for a meaningful analysis, as anticipated by the reviewer, so we have not commented on it in the manuscript.
The presence of a exonuclease activity in the viral DNA polymerase seems to be associated to the low mutation rate observed on baculoviruses. There is a recent work (Aguirre et al. Viruses 2021) showing that in an alphabaculovirus, one variant is able to generate high levels of variation. This is not the first time that high variation is observed in baculovirus populations. Although this observations do not hampered the experimental results, they must be taken into account when explaining how variants are generated and maintained in baculovirus populations.	We thank the reviewer for this thoughtful suggestion. In the concluding remarks paragraph we have now mentioned that mutator phenotypes occur in natural baculovirus populations, and that they may indeed play a role in generating variation (page 19).
I think this point should be mentioned, as in previous papers there are references to "hot spots of mutations".	All of the reviewers rightly point out that mutations are not expected to be distributed uniformly across the genome. We have now analyzed the distribution of mutations in the bacmid insert and viral genome. We found that neither are uniformly distributed, supporting the idea that the occurrence of mutations is indeed dependent on the genetic context (structure, sequence). We have included these results (Table S1) and mentioned the distribution of mutations in the Results and Discussion (page 8).
The authors analyze the robustness of their model by a sensitivity analysis, but only of some of the parameters. It is interesting that the replication mechanism (strain copying or rolling circle) does not change the mutation rate estimates. What about the value of kappa, that is, the size of the virus population on a given host. Has a sensitivity analysis been performed using various estimates of the population size. Clearly the number of genomes present in the OBs is a subset of the total number of genomes replicated. It does not take into account the genomes that for BV not those that could be not included in nucleocapsids. It could be expected that the subset encapsidated into the is chosen randomly, but this could be not the case as OB production occurs at later times of infection, and thus the cellular machinery of repair might be not in optimal conditions.	The model we have developed requires considerable computational resources to generate model predictions, and we therefore had to limit the scope of the sensitivity analysis to parameters for which we have no empirical estimates to keep the analysis feasible. (I.e., the simulations for the updated model took more than two weeks to run.) However, the reviewer raises an interesting point concerning the effect of bottleneck and final population size in larvae, and we decided to look into this. We have now run the fitted model for a range of population sizes and bottleneck sizes and looked at the number of detectable mutations (above the threshold frequency of τ) that accumulate, as a first exploration of the effects of these parameters. We find that both parameters affect mutation accumulation, stressing the importance of (1) using models that [...]

	[...] incorporate demographic processes, and (2) having solid parameter estimates, for estimating mutation rates. We have briefly summarized these new findings in the Results and Discussion (page 15), and given a full description in the supplementary materials (Appendix Text 1, Figures S5-S6).
In a similar way, the multiplicity of infection, that is, the value of parameter Lambda, has been fixed to 46, due to the mode of infection of the larvae. The authors have fixed the numbers of ODV by OB, and fixed the number of nucleocapsids per ODV, but these values are very variable, even within a single production. The distribution of nucleocapsids par virion does not follow a normal law. A high number of ODV have a single nucleocapsid. Can the authors speculate on the consequences of varying this parameter, as the final number of genomes entering into the larvae is not known?	We failed to explain that our simulation model already incorporated variation in the number of founders. In the original version of the simulation model, Lambda is the mean of a Poisson-distributed random variable. In retrospect, we agree with that variation in Lambda will be larger: The number of infecting ODV can reasonably be assumed to follow a Poisson distribution, but the number of nucleocapsids per ODV will then add extra variation to the number of infection founders. For the AcMNPV bacmid, the distribution of nucleocapsids per ODV is similar to a Poisson distribution with a mean ~4 (Zwart et al. 2008). In the adapted model, we therefore have a Poisson distributed number of ODV per larvae (with a mean Lambda / ~4), and zero-truncated Poisson distribution with a mean ~4 for nucleocapsids per ODV. This leads to higher levels of variation in the number of founders than in the original model, and is now reflected in our mutation rate estimates (although the effect is probably minimal, as the ODV bottleneck is not very narrow). The adapted methods and parameters are described in the Materials and Methods section (page 24) and Table 4.
Line 132. What does it means "per strand copying", the number of replication cycles in each insect is not known.	We assume there is exponential growth of the initial population (with mean lambda) until the carrying capacity kappa is reached, with the (variable) factor rho describing the mode of replication. We explain our rationale for the model (pg. 23) and have now added a brief discussion of this choice (page 20).
Line 154; What means "viral generation?	We should have been more precise and called this the "mutation rate per base per strand copying", and this mistake has been fixed (page 12).
Line 193. As you provided evidence that nonsynonymous mutations are cleaned from the genomes, why not to use the synonymous mutations only in viral genes when calculating the mutation rate? This could help on the analysis of the transition to transversion ratio (line 236).	This is an interesting and thought-provoking suggestion. However, transitions are more likely to be synonymous than transversions, due to structure of the genetic code. Hence the synonymous mutations observed will be heavily biased towards transitions. To avoid these and other subtle biases (i.e., in practice synonymous mutations are often not entirely neutral), we have chosen to only analyze the mutations in the bacmid for the formal mutation rate estimates.
Line 303. The value of Lambda should be -10*ln(1-0.99) to get a positive value.	Thanks to the reviewer for spotting this error, which has been corrected (page 27).

Reviewer 2	
One biological context that goes unmentioned in this study is the matchup of the particular AcMNPV BAC clone under study, and the Spodoptera exigua (beet armyworm) larvae used as the experimental evolution host. Presumably there was a rationale for choosing this isolate and this host – can the authors please add this? Might the choice of host be influencing the level of selective pressure on the virus? The discussion is extremely short, so there is room to explain how these choices may have influenced the outcome.	We thank the reviewer for raising this relevant point. We chose this host-virus pair based on (1) the availability of an infectious clone that could be introduced straight into insects, (2) having a colony which in which AcMNPV infection does not activate latent viruses and thereby lead to contamination of evolved Bacmid lines, and (3) having a host colony which is highly susceptible to the virus. We have now provided this motivation and discussed the implications of the host species (page 21-22). Overall, the “concluding remarks” section has been expanded.
Another biological context that goes undiscussed is the choice of an extremely high MOI inoculum – despite the low stated bottleneck of 46 – and the experimental design that pools billions of viral particles across multiple larvae cadavers, to create the next round of viral inoculum for each lineage. This approach seems sure to reduce the impact of any rare variants, and does not seem likely to reflect natural environmental bottlenecks. This leaves the impression that while the mutation rate can be as low as that measured here, the natural situation may be more varied.	The combination of (i) an inoculum with a relatively large number of virus particles, and (ii) a relatively narrow, dose-dependent bottleneck, is the norm for a most virus and multicellular host pathosystems. There is a very large body of published work documenting these patterns in many different virus-host systems. In our setup, starved larvae are exposed to a high OB concentration, and the imbibed volume will result in a large dose of occlusion derived virions (ODVs) being released in the midgut. However, the probability of infection per ODV is small, so only a small number will establish infection in cells lining the midgut, and many of these infected cells be sloughed off before infection can spread. Hence, despite the high dose, the number of virus individuals causing infection (the bottleneck size, Lambda) is small. The difference in the census size of the inoculum and the number of founders is driven by the difficulty for the virus to infect the host, and as we follow a natural infection route, it is very representative of what happens in the real world. Population genetics studies have shown that bottlenecks in the field also tend to be narrow (E.g., Kennedy and Dwyer, 2018). Although most viruses don’t have a biphasic virus-particle morphology (i.e., ODV), the general principle is the same: you typically require large doses for infection, but the infection bottleneck is narrow. We now briefly mention this point and provide some references to point readers to this large body of work (see Table 3).<br><br>We fully agree with the reviewer that demographic conditions will affects patterns of mutation accumulation, and it is precisely for this reason that we have included such a detailed simulation model in our mutation rate estimates. In our revision, we now document how some of these demographic parameters will affect mutation accumulation (Appenddix Text 1, and Figures S8 and S9; see also our response to the final major point of Reviewer #1). Finally, we don’t see a mechanism whereby the pooling of OBs from different larvae would fundamentally change the pattern of mutation accumulation or make them more difficult to predict, other then increasing the overall population size. Clearly the multiple larvae should be taken into consideration as this matters for mutation accumulation, but that is precisely why we include all these conditions in the model for mutation rate estimates from the beginning.

Two other aspects of the natural biological context for these viruses are standing genetic variation in the population, and the ability of coincident viruses to undergo recombination. The authors have intentionally aimed to use a homogeneous source population, but these other contributions impact the natural polymorphisms observed in isolates of AcMNPV at least belong in the discussion. At present, the authors leave it thus: "By contrast, the high genetic variation often observed within alphabaculovirus populations [21-24] remains a conundrum." The biological contexts listed here, among others, would help to explain this conundrum.	We thank the reviewer for these suggestions to balance the discussion, which we both consider in the discussion now (page 19). Due to population bottlenecks that virus populations experience, standing genetic variation will be lost rapidly and another evolutionary force (mutation or selection) is required to have high levels of polymorphism.
Throughout the text, there is an over-emphasis on comparing the authors' work to data, methods and controls that have been necessary in prior studies of small RNA virus genomes. The vast majority of literature cited for comparison is likewise on (very excellent) RNA virus studies. Yet these are not the best comparisons for the present study, and this emphasis is likely to confuse the reader. In the introduction, the authors posit that "most mutations in viral genomes are deleterious", with citations to RNA virus studies. Large DNA viruses have sufficient intergenic space, genetic redundancy, repetitive elements, etc. that this claim is not likely to be correct for these viruses. This claim should be removed or backed up with relevant DNA virus references. As a methods example, the CirSeq approach used as a comparison on line 270 is an approach developed for RNA viruses to counterbalance for the need to use (error-prone) reverse transcriptase to create DNA templates from the initial pool of RNA virus genomes. That biological step is not necessary for baculoviruses, so this is not a useful contrast to make with the authors' methods here. It would be more useful and appropriate to compare the present work to recent in-depth genomics analyses of adenoviruses, poxviruses, herpesviruses, megaviruses, etc.	The argument about the prevalence of deleterious mutations was made in the context of adaptation and the scarcity of beneficial mutations. We agree that the distribution of mutational effects could be different for large DNA viruses, and have reworded our statement accordingly(Page 4).<br><br>The reviewer is of course correct that in practice duplex sequencing is used with dsDNA viruses, and we have modified our text accordingly (page 19). (Although we note Raul Andino himself makes clear that main motivation for CirSeq was to mitigate Illumina sequencing errors - see for example J Virol 90: 8950 –8953.)<br><br>We thank the reviewer for pointing out there were some noteworthy approaches and results for dsDNA viruses that were not discussed, and we have now amplified the discussion to include these (page 20). Note that the adenovirus mutation rates (Risso-Ballester et al. 2016) cannot be directly compared to other dsDNA virus estimates because of the units (mutations per site per round of cellular infection), but we now mention this result.
The comparisons in the manuscript focus on the mutation rate of the AcMNPV genome, as compared to the "neutral intergenic region" of the BAC-insertion. While this is interesting, it seems odd to not include any mention or analysis of the natural intergenic regions of the AcMNPV genome. Since the authors calculated dN/dS, their analysis pipeline includes an awareness of gene-encoding regions and the intergenic areas between them. At present it is not stated how these are handled. Did the mutations observed in this study all fall into coding regions? Or were they predominantly intergenic? These data should be included, and the natural AcMNPV intergenic regions should be analyzed as a group for comparison, alongside the artificial neutral-intergenic (dI) region of the BAC, and the dN/dS rate of the rest of the AcMNPV genome.<br><br>Minor comment: What is observed around the homologous regions (hrs) of this baculovirus genome? Does the alignment approach (mapping to the reference genome, line 308) obscure the ability to observe any fluctuations at these repetitive regions?	We thank the reviewer for this thoughtful comment. There were a considerable number of (typically low-frequency) mutations in AcMNPV intragenic regions, as documented in the supplementary materials in the original submission. We have now mentioned these results in the main paper, and followed the reviewer's suggestion to calculate the dI/dS for the viral genome. With the lowest mutation detection threshold (Tau = 0.5%), there is a strong over-representation of intergenic mutations, whereas there are none or very few for the highest mutation detection threshold (Tau = 2%). Most of these intergenic mutations are indeed associated with the homologous regions, which are likely to be prone to sequencing error but may also represent mutation hotspots. We therefore cannot draw any conclusions from this analysis. We have now mentioned the intergenic mutations in the results section and added the *dI/dS* analysis for the natural virus genome (page 14 and expanded Figure 3).

It is not clear why the authors chose to exclude the sizable number of mutations that occurs in the ancestral BAC, and any that showed up in more than one progeny virus. While the text (lines 313-316) suggests that this was done to exclude any contribution of variants in the initial starting population, this seems like an odd choice – since natural virus populations may have this much or an even larger amount of variation. The choice to exclude variants that appear more than once likewise seems poised to exclude the very real possibility of mutational hotspots in the viral genome.	In this study, we estimate a mutation rate: how often *de novo* mutations occur over a given period of time. Hence, it follows that all mutations must occur during the experiment, and not before it. Mutations detected in the ancestral bacmid did not occur during the experiment (or possibly are artefacts), and hence they must be excluded from the analysis. This is a *sine qua non* condition for any mutation rate analysis (see e.g., Sanjuán et al. 2010).<br><br>Mutations that are found in multiple lineages are suspect, because (especially for the bacmid region) we working in a setup in which we have ruled out selection, by and large. Note that even in the presence of strong selection and large effective population sizes, observing the exact same mutation in a majority of lineages is rare (see for example references 52 and 53). One would need to invoke very strong mutation bias to explain why so many mutations are occurring in multiple lineages. We think this explanation is implausible, and it is much more reasonable to assume that these mutations are sequencing or read-mapping artefacts that were not detected in the bacmid (and in most cases, probably close to the detection limit in our pipeline). Not being conservative in mutation calling has clearly led to erroneous conclusions in deep-sequencing studies (see for example Sci. Transl. Med.13, eabh1803).<br><br>Also note that the effect of filtering for sequence read quality (coverage, forward reverse balance, etc) has a greater effect on the number of mutations found in each lineage compared to the maximum number of parallel lineages in which we allow a particular mutation to occur. The filtering decreases the number of mutations found by between 88-94% for each of the maximum number of lineages chosen (1-5), The maximum lineages allowed affects the estimate of the number of mutations by an increase of approximately 20-50% for each additional maximum (ranging from 1 to 6).<br><br>Although we are firmly convinced that filtering repeated mutations is the best practice, we have no objections to considering how this assumption effects mutation rate estimates and being transparent about this in our presentation. For this reason, in the analyses throughout the paper we have now considered the effects of filtering repeated mutations with different levels of stringency (i.e., setting a threshold number for the maximum number of lineages in which a mutation can occur, denoted by variable psi) throughout the analyses presented (E.g., see Tables 1 and 2, and Figures 2 and 3). We also clearly present our rational for choosing to filter repeated mutations (pages 7-8 and 20-21).

line 265 - this approach is not particularly novel. There is a long and classic literature using the insertion of exogenous sequences into viral genomes (and many other species), and using various methods to observe the mutation rate of the inserted sequence. Recognition and citation of this history would be more appropriate, and then the authors can distinguish how their application of this deep sequencing and this particular host-virus matchup provides novelty.	The reviewer is correct that using neutral regions for mutation rate estimates (in at least one case even in combination with high-throughput sequencing) is not novel. We have rephrased this paragraph to better reflect what is new in our work (pages 19-20).
What isolate or lineage of AcMNPV is contained in pBac-E2? Was this resource generated for this study, or is there a prior citation or description of its construction? These details should be referenced or included.	In the original paper, we gave background information and a reference at the start of the results and discussion section. However, we recognize that readers who want more information on the setup may jump to the methods section, and we have expanded the information provided there and repeated the reference to previous work (page 22).
How were the library preps generated for HiSeq sequencing? Presumably PE150 indicates paired-end reads?	We have included information on the library preparation and removed any ambiguity about the sequencing approach used (page 23).
Why not include other large DNA viruses in figure 4? There are relevant comparative data on from similar studies of adenoviruses, poxviruses, herpesviruses, megaviruses, etc. These should be included as well.	As pointed out in the text, this table includes only experimental estimates of the mutation rate per site per strand replication event (s/n/r), to make the comparison rigorous. The dataset is based on an overview made by Rafael Sanjuán, and we have updated estimates when these are available. The adenovirus and herpesvirus mutation rate estimates are “mutations per site per round of cellular infection” estimates, and therefore cannot be directly compared (Sanjuán et al. 2010). For the poxviruses and giant viruses, we are not aware of comparable experimentally based estimates of mutation rates.
What reference genome was used here, for mapping of all data (line 308)? Does it have an accession number in a common repository (EBI/GenBank)? It is not stated if it is derived from exactly this isolate, or if not, how closely it matches the BAC.	We had previously included the reference genome in the supplementary materials. This variant does not have an accession number, and as it is a variant engineered for a specific purpose we don’t think it makes sense to submit it to a general sequence database. We now specify were this sequence comes from (page 23).
Figures S1-S3 could easily be combined into a single figure.	As there is a large number of supplementary figures and the number of mutations is very small for higher vales of the mutation detection threshold, we have opted to only give a version of this figure for the lowest mutation detection threshold (τ = 0.5).
Sequencing data should be deposited in a common repository such as the Sequence Read Archive.	Good suggestion - we have deposited the raw sequence data and provide a link in the paper (page 23).
Reviewer 3	
L 109: Isn’t the viral amplification step in L3 a 6th passage in the experiment. Please explain the possible impact this amplification passage could have on the parameters used for the models to estimate the mutations rates	We thank the reviewer for this helpful comment. Indeed, although the conditions for passaging are different (larval development stage and number of larvae), this step can and should have been taken along in the model. In the revised manuscript, we expanded the simulation model and classical [...]

	[...] methods to include this step (page 26) and therefore take its impact on mutation rate estimates into consideration now. This extra passage results in a slight reduction in the mutation rate estimate for all methods, as the number of replications events is increased.
L113: Around there indicate the average sequence 5500 coverage for your genome. It is much lower than in previous deep sequencing analyses done on AcMNPV.	Additional coverage will only help until a certain point. Our coverage is on average much higher than the Illumina error rate, so additional sequencing coverage would not have made it possible to detect mutations at lower frequencies. I.e., for a thousand-fold higher coverage we would have used the same threshold values for mutation frequency.
L258-259: In the conclusion it would help readers to estimate how many neutral mutations per genome (and per OB) could be transmitted to the next generation given the actual calculated rate. This could be compared this with the number of transposable elements found in AcMNPV OBs.	In response to this suggestion, we considered different ways to make some intuitive and credible predictions. However, in the revised manuscript we now argue explicitly that demography will determine accumulation of strictly neutral mutations. Given that we accentuate this dependence, it would be incoherent to make a single prediction for the rate at which mutations accumulate. The simulations now included (Figs S5-S6) should give readers an impression of the rate of mutation accumulation over a range of demographic conditions.
L286: What was the volume of the droplet used for the droplet feeding assay. (How many OBs do the caterpillar ingest?). How does this relate to the genome bottleneck size of 46?	For the serial passage experiment, we only know the OB concentration and not the ingested volume. Our estimate of population bottleneck does not depend on the exact inoculum dose, but rather on the relationship between host survival and the bottleneck size. We hypothesized and experimentally confirmed that the size of the bottleneck can be predicted by the fraction of surviving hosts (Reference 41), and that for AcMNPV infection of early instar *S. exigua* larvae this relationship is dose-dependent.
L291: What was the OB concentrations and volumes used of infect the L3 caterpillar in the 6th (amplification) cycle? Was the final size of the virus population per insect cadaver still 5.32x108? In L3 the yield should be higher than in L2.	We have now included the details in the manuscript (page 22). We did not measure the OB yield per larvae for L3 here, and so we assumed the final population size was twice the value for L2. As this is the final round of amplification, this parameter has virtually no effect on mutation accumulation. (I.e., *de novo* mutations which occur late in infection [i.e., as the census population size approaches κ] will be below the detection threshold of mutations – for which the lowest value is τ = 0.5%.)
L312-317: I don't understand why convergent evolution of mutation in different lineage should be excluded as possibly deriving from polymorphism in the BAC population. Is this an indication of insufficient sequence coverage for the experiment?	We refer the reviewer to the final major issue raised by reviewer 2 (on pages 4 and 5 of this reply to reviewers), which raises the exact same question. In brief, we think there are many good reasons why mutations that are found in multiple lineages are much more likely to be sequencing errors than repeated events. While we argue this is the case in the revised manuscript (pages 12, 20 and 21), we acknowledge that we cannot know for sure. We therefore have introduced a new variable in our analysis, the number of lineages in which a mutation can occur before we filter it out (ψ). We now [...]

	[...] perform all analyses for ψ values of 1 (no repeated mutations), 3 and 5 (all repeated mutations included), so that the reader can consider the impact of this assumption (see Figures 2, 3 and S4, and Tables 1, 2, and S1-S4). As ψ is increased, the estimated mutation rate increases. Over all conditions, the highest mutation rate estimate obtained was 5×10^{-7}, which represents a moderate increase compared to what we consider the most conservative estimate (5×10^{-7}).
L 320: Would the sequencing error rate in the illumina data allow to use a lower mutation threshold given the sequencing coverage?	We trimmed the sequencing reads such that the probability of a sequencing error is 1:1000 (Phred score of 30). However, in practice sequencing errors can be more abundant and context dependent, so we think having a threshold that is minimally 5 times the expected error rate is prudent.
L330-332: what would it change if reversions are allowed?	The fraction of mutated sites in evolved populations is very small (i.e., < 1:1000 and in most cases much lower), so the probability that a new mutation further modifies or reverses a mutated site is negligible here.
L387-389: Throughout the material and methods the authors assume mutations are extremely rare and seem to exclude all data and parameters that would increase the calculated rate.	We object to these remarks in the strongest possible terms, as the reviewer is questioning our scientific integrity. When this particular case (Lines 387-389 in the original submission, page 20 in the revision) mentioned is examined in detail, we believe it convincingly exemplifies the exact opposite of what the reviewer has claimed: we have approached the analysis with an open mind and scientific rigor.<br><br>In this section, we describe an alternative method to calculate the mutation rate, which we performed as a check for our simulation-based method. Two changes are described, and both were made for very good reasons:<br>(1) Not including the correction for selection: All indications are that mutations in the bacmid region are neutral.<br>(2) Removing the factor 3 from the numerator: The classical method was developed for Sanger sequences derived from clones, so for each locus (i.e., nucleotide position) it is only possible to identify one of the three alternative nucleotides which could occur, and this bias needs to be corrected for (and hence the factor 3). With high-throughput sequencing of populations, different mutations that occur simultaneously at each locus can be identified concurrently, so the correction factor should be removed from the calculation.<br>If we had not made these two changes, the estimated mutation rate by this alternative method would indeed be higher. *However, this alternative method gave a considerably **lower** estimate of mutation rate than our simulation-based method!* Had we been biased, we would have simply used the unmodified alternative method and reported results that are closer to our simulation-based results. But we choose the route of integrity: using the best approach even if the result was not convenient.
